# An evaluation of a multi-site fetal alcohol spectrum disorder models of care project

**DOI:** 10.3389/fpubh.2023.1195484

**Published:** 2023-07-24

**Authors:** Kirsten R. Panton, James P. Fitzpatrick, Carmela F. Pestell

**Affiliations:** ^1^School of Psychological Science, The University of Western Australia, Perth, WA, Australia; ^2^Patches Assessment Services, Subiaco, WA, Australia

**Keywords:** fetal alcohol spectrum disorder, prenatal alcohol exposure, FASD, training, diagnosis

## Abstract

Fetal alcohol spectrum disorder (FASD) continues to be underdiagnosed in Australia, partly due to the lack of trained clinicians and diagnostic services. This project aimed to help increase FASD knowledge and diagnostic capacity across Australia. Six sites across Australia formed part of a national consortium, delivering training clinics, diagnostic clinics and community education sessions. The number of FASD diagnoses significantly increased across the project. Additionally, the number of community education sessions steadily increased across the project, with largely positive feedback. Participants attending the training clinics demonstrated increased knowledge of and confidence in FASD diagnosis. This evaluation showcases the benefits of a coordinated approach to prevention, assessment, diagnosis and training in FASD.

## 1. Introduction

Fetal alcohol spectrum disorder (FASD) is a diagnostic term that captures the neurodevelopmental and physical impairments resulting from alcohol exposure *in utero* (1). FASD continues to be underrecognized and underdiagnosed (2, 3), with general prevalence estimates between 0.01 and 0.68 per 1,000 live births in Australia (4, 5). However, the current prevalence rates are considered to be underestimates, as we have largely relied on passive surveillance systems to monitor FASD (4). Several studies have also attempted to estimate the prevalence of FASD in specific populations in Australia through active case ascertainment, with higher prevalence rates found in these sub-populations (6, 7).

The Australian FASD diagnostic guide was released in 2016; however, we have long since known about the potential harm of alcohol on the developing fetus (8–10). It has taken time for FASD to gain recognition in Australia, though we have seen positive movement toward change, with the Department of Health releasing the 2018–2028 National FASD Strategic Action Plan (11). The Action Plan (11) advocates for activities centered around FASD prevention, screening and diagnosis, and support and management.

FASD tends to be recognized as a diagnostic term, though individuals working closely with FASD (including health professionals) are often less familiar with the common behaviors associated with FASD (12–15). Similarly, individuals in the general public are likely to have heard of FASD but are less clear on key characteristics (16, 17). Even though it is promising that FASD continues to grow in recognition, in order to prevent, diagnose and manage FASD, it is important that both the general public and health professionals have a better understanding of FASD.

There continue to be significant barriers to obtaining a FASD diagnosis, and through this project, we aimed to reduce some of the existing service-level barriers (18, 19). One of the major issues is a lack of appropriately trained clinicians (20, 21). Clinicians are also fearful to ask about alcohol use during pregnancy due to concerns around stigma and the emotional burden it may bring for the parent or caregiver [e.g., blame, guilt, anger (18, 22)]. However, without a diagnosis, individuals will have difficulty accessing the appropriate supports to best meet their needs (23).

The current study examines the success of a national FASD prevention, assessment and diagnosis effort from 6 sites across Australia. Each site conducted community education sessions (CES) and FASD training clinics for health professionals to build relevant FASD knowledge. Additionally, each site assessed and diagnosed FASD within multidisciplinary (MDT) clinics. It was predicted that the number of FASD diagnoses would increase from the start (i.e., before these FASD clinics were established) to the end of the project. Similarly, the number of community education sessions and attendants was expected to increase as the consortium sites built their local FASD networks. Finally, it was predicted that individuals would see an increase in knowledge and diagnostic confidence following FASD-specific training.

## 2. Methods

### 2.1. Project design

This project had two broad aims of increasing diagnostic capacity and increasing FASD awareness, knowledge and advocacy through various activities. This paper evaluates the outcomes of the training and prevention activities and diagnostic service outcomes. The accompanying qualitative study describes the process of opening five new FASD MDT clinics and developing local models of care (24).

#### 2.1.1. Sites

There were 6 sites involved from across the country, including Patches Assessment Services [Western Australia (WA) and Northern Territory (NT)], Central Australian Aboriginal Congress (CAAC; NT), Danila Dilba Health Service (DDHS; NT), Child Development Unit, Women’s Children Hospital [CDU WCH; South Australia (SA)], Goulburn Valley Health Service [GVHS; Victoria (VIC)], and FASD Tasmania [Tasmania (TAS)]. The University of Western Australia Site Lead and Project Officer led the evaluation of the project (authors CP and KP respectively).

#### 2.1.2. Diagnostic and training clinics

Diagnostic activity was tracked with the number of clients assessed, number of clients diagnosed with FASD and number of clients diagnosed with FASD that identified as Aboriginal and Torres Strait Islander. There was some discrepancy in the reporting of Aboriginal and Torres Strait Islander clients, with some sites reporting the total number assessed, and others reporting the total number diagnosed with FASD. Additionally, the number of clients assessed was a diluted metric, as some sites (e.g., CAAC) assessed a range of neurodevelopmental disorders (e.g., ADHD, ASD).

At each site, diagnostic training clinics were delivered to local clinicians to help upskill and build local diagnostic capacity. All clinicians within the project received direct training from experienced practitioners in FASD assessment and diagnosis, Dr. Pestell (clinical neuropsychologist), and Dr. Fitzpatrick (paediatrician). The training included structured training in-person across multiple days, covering all aspects of FASD assessment and diagnosis, namely: effects of prenatal alcohol exposure on the developing fetus, prevalence and patterns of FASD in various populations, medical aspects of assessment (diagnostic history, ascertaining PAE, growth assessment, facial feature assessment, physical assessment), and comprehensive training in the assessment of the ten brain domains within the framework of the Australian Guide to the Assessment and Diagnosis of FASD. Training often included the opportunity for direct observation of clinical assessment through regular training clinics, where trainees observed the testing and assessment, formulation and case conferencing, and report writing aspects of a comprehensive multidisciplinary FASD assessment. Training clinic activity was measured by the number of training clinics, number of clinic attendees and attendee profession.

#### 2.1.3. Community education sessions

Each site was expected to deliver at least eight CES annually to help build FASD knowledge and awareness in local communities. CES activity was measured by the number of CES and number of CES attendees. The CES were primarily delivered by the Site Coordinator, which had varied clinical backgrounds, including nurse/midwife (*n* = 3), social worker (*n* = 1) and occupational therapist (*n* = 1). The CES broadly covered FASD prevalence, FASD diagnostic features, the importance of FASD diagnosis and FASD intervention.

### 2.2. Participants and measures

#### 2.2.1. CES and training clinic feedback

A proportion of training clinic participants (*n* = 273) and CES participants (*n* = 621) provided formal feedback for the sessions. The CES and training clinic feedback forms were primarily distributed in the early stages of the project and prior to the COVID-19 pandemic. Although participants provided some qualitative feedback which was used to improve the training clinics, only quantitative feedback was approved to be used for research purposes. The four questions were: (1) “What is your overall assessment of the session”?; (2) “Were the learning objectives clearly stated”?; (3) “Did the knowledge and information gained from participation at this session meet your expectations?”; and (4) “Will the information covered be useful/applicable in your future clinical practice”?. Questions were rated from 1 (insufficient) to 5 (excellent). Questions 2–4 were coded as “definitely” = 5, “mostly” = 4, “somewhat” = 3 and “not at all” = 2. This coding was chosen to match most closely to the coding in question 1. The training clinics were mostly attended by pediatricians (*n* = 72), clinical neuropsychologists or psychologists (*n* = 69), speech pathologists (*n* = 31) and nurses (*n* = 29). Several clinics also hosted students from psychology (*n* = 16) and medical (*n* = 13) disciplines. In comparison, CE sessions were mostly attended by community workers, including education/childcare workers (*n* = 81), nurses/midwives (*n* = 74), social workers (*n* = 60) and speech pathologists (*n* = 40).

#### 2.2.2. FASD confidence and knowledge survey

For a small proportion of training clinic participants (*n* = 21), a pre-and post-knowledge survey was administered, which included a more qualitative and quantitative appraisal of their FASD knowledge. The first portion of the survey asked participants to rate their confidence in FASD knowledge (e.g., “I have a good understanding of the cognitive features of FASD”) on a five-point Likert scale (“strongly agree” to “strongly disagree”). The second part of the survey tested participants’ specific knowledge of FASD with forced multiple choice (e.g., “The three sentinel facial features are”), multiple answer [selecting all that apply, e.g., “According to Australian diagnostic guidelines for FASD, which of the following are considered evidence of severe impairment in affect regulation? (select all that apply)”] and true or false (“There is no known safe level of alcohol consumption during pregnancy”) questions. For the training clinic, there were 15 confidence and 10 knowledge questions (see Appendix 1). The questions were identical from pre-training/course to post-training/course. The training course participants completed the questionnaire directly before and after the training clinic.

The training clinic participants were primarily clinical neuropsychologists (including one registrar; *n* = 18), with other participants identifying as clinical psychologist (*n* = 1), postgraduate psychology student (*n* = 1) and undergraduate psychology student (*n* = 1), with an average of 14.05 years of clinical experience across participants (SD = 11.09 years). A proportion of the neuropsychologists also identified as clinical psychologists (*n* = 5) and forensic psychologist (*n* = 1).

### 2.3. Procedure

Ethics approval for this project was obtained through The University of Western Australia (RA/4/20/5792). Diagnostic services were launched at each site further details can be found in (24). Each site was asked to distribute feedback surveys at the end of each training and CES. Each site was required to report on its activity quarterly from August 2017 to August 2020. The lead researcher entered the relevant data into a master spreadsheet, tracking activity for each site over the government reporting periods for the project (6 monthly).

### 2.4. Data analysis

#### 2.4.1. Diagnostic clinic, training clinic and CES

The diagnostic clinic, training clinic, and CES data were organized by six 6 monthly periods for each site. Due to the inconsistency in the reporting of training clinics by each site, it was difficult to meaningfully compare this data across the project, and as such, no formal statistical analyses were conducted on this data.

The diagnostic clinics and CES data were non-normally distributed and as such were analyzed using non-parametric tests. The Wilcoxon signed-rank test was chosen to compare data at two different time points (i.e., the start and end of the project). As most sites had to cancel or reschedule diagnostic clinics, training clinics and CES, the 6 months period pre-COVID was chosen as the final comparison point. As Patches was an established clinic, it was not included in the diagnostic activity data.

#### 2.4.2. Feedback

The feedback data is presented as frequencies, as this data was only collected at one time point.

#### 2.4.3. Knowledge surveys

The knowledge and confidence survey data were normally distributed, and were analyzed using paired samples *t*-tests, comparing FASD knowledge and confidence before the start of training and after training.

## 3. Results

### 3.1. Diagnostic activity

**Figure 1:**
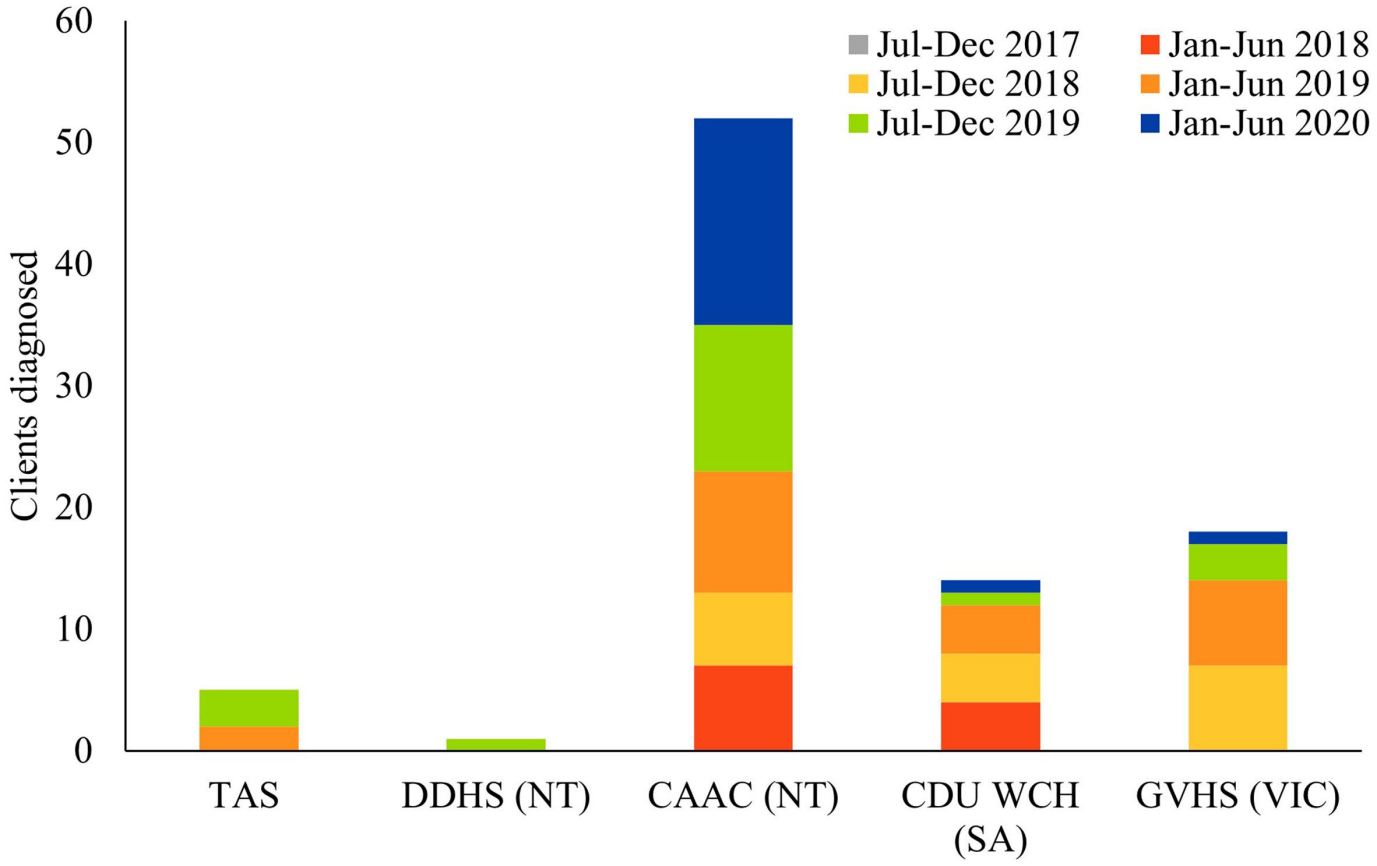
This figure represents the number of FASD diagnoses made across the 6 reporting periods for each state (excluding Patches WA & NT).

The number of individuals diagnosed with FASD was used as the metric for diagnostic success at each site. By rolling out FASD diagnostic clinics across the country, Wilcoxon signed rank tests revealed that diagnostic activity significantly increased from the first reporting period (July 2017–December 2017; *M* = 0, SD = 0) to the final (pre-COVID) reporting period (July 19–December 19; *M* = 4.00, SD = 4.58), *Z* = −2.06, *p* = 0.04 (Figure 1). Patches (WA & NT) was not included in this analysis, as Patches was already an established FASD diagnostic clinic.

### 3.2. CES

**Figure 2:**
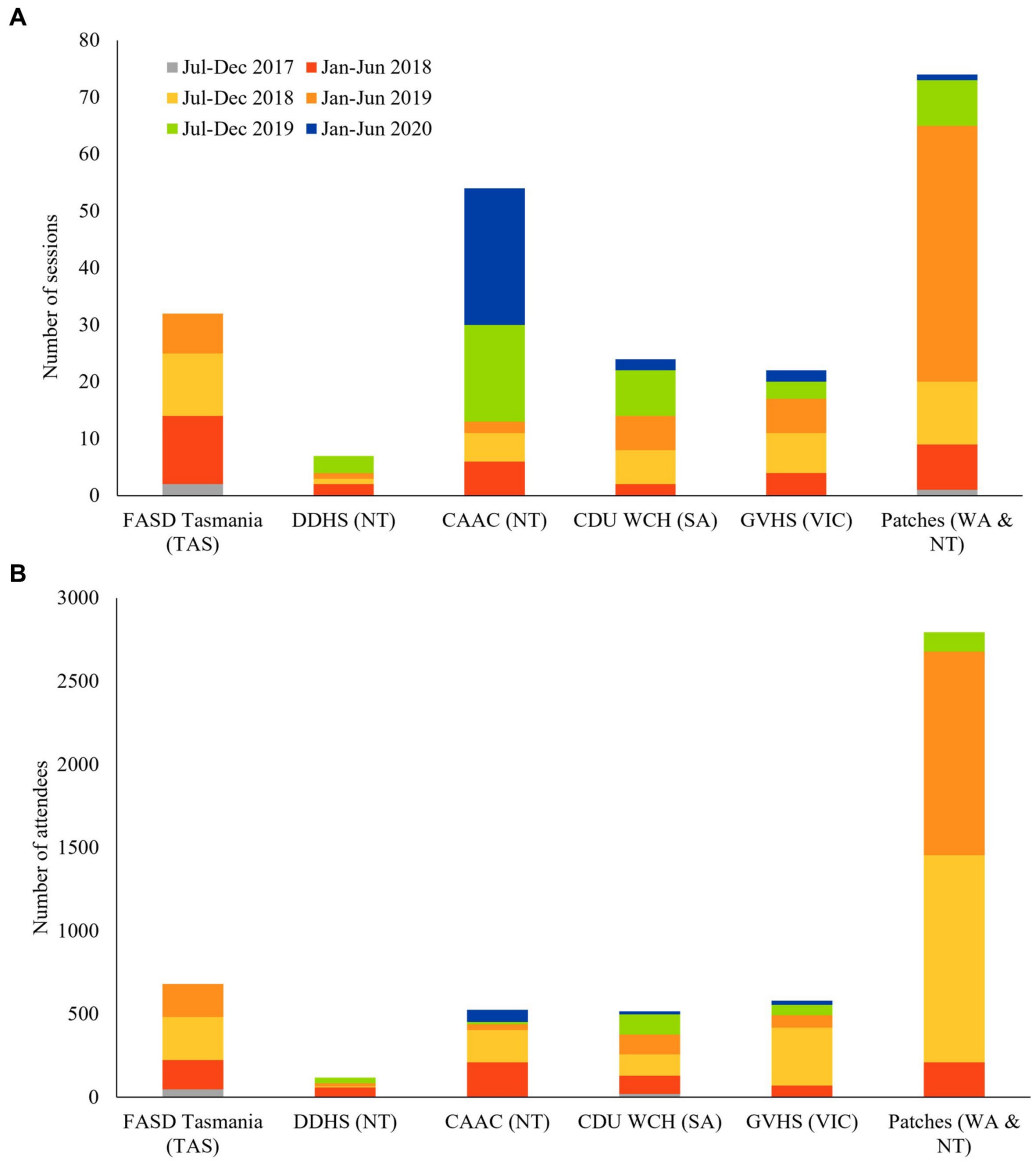
Number of CES **(A)** and attendees **(B)** per site.

There was a steady increase in the delivery of community education across the project, however, there was a notable decrease in the delivery of CES in the final reporting periods (Figure 2). This is partly attributable to the global pandemic, which saw some sites cancelling sessions rather than opting for online alternatives. However, Wilcoxon signed ranks test revealed that the increase in CES sessions was not statistically significant when comparing the first reporting period (*M* = 0.40, SD = 0.89) compared to the final (*M* = 6.20, SD = 6.89) reporting period, *Z* = −1.75, *p* = 0.08.

### 3.3. CES and training clinic feedback

**Figure 3:**
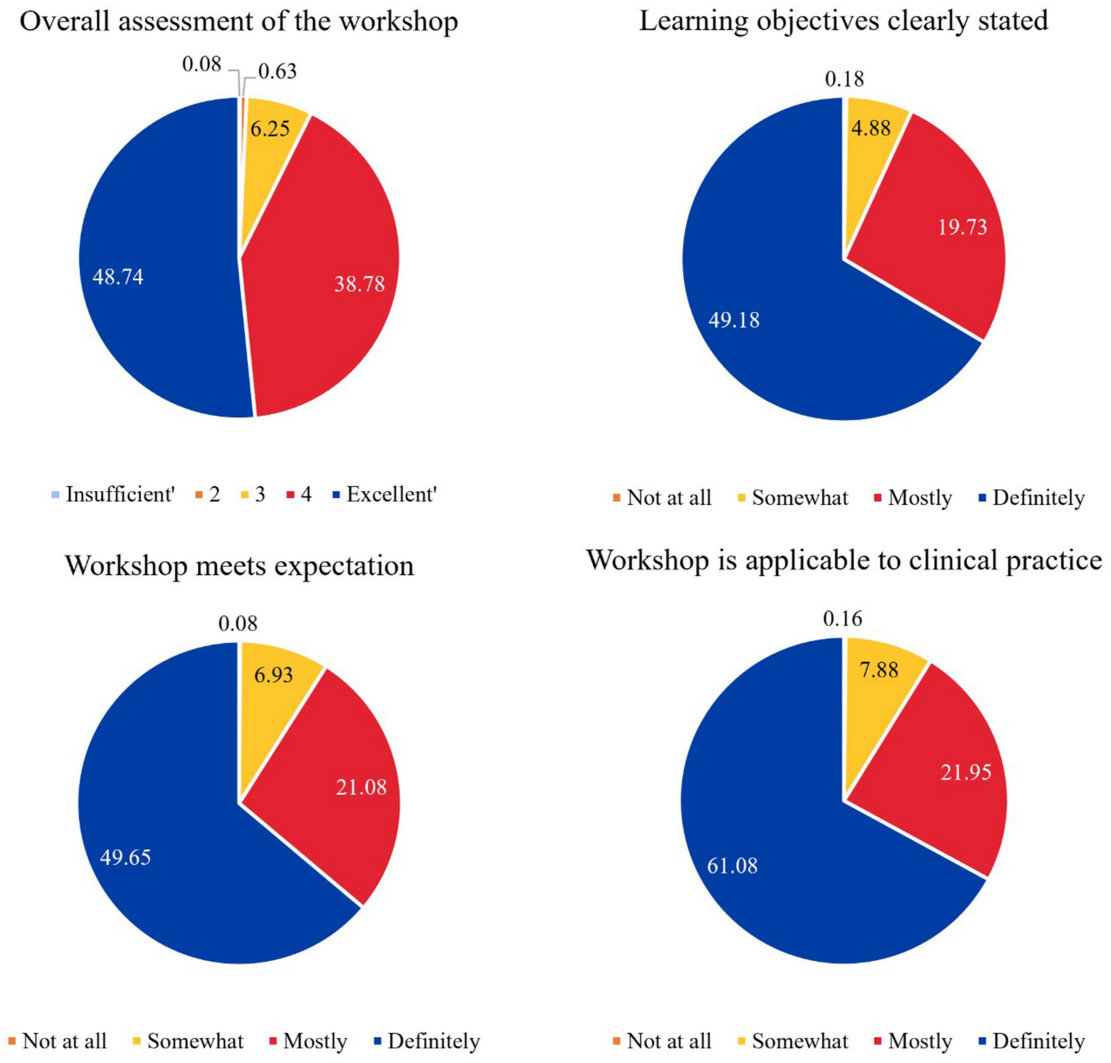
Participant feedback on training clinic and community education sessions.

Feedback was obtained from a small portion of participants attending the community education and training clinics, as most sites stopped administering feedback forms after the first two reporting periods (Figure 3). Of these participants, most rated the sessions as “excellent” (4 or 5, 87.51%), meeting the learning objectives (68.91%), meeting expectations (70.73%) and applicable to clinical practice (83.03%). There was missing data on each of the items, as follows: “overall assessment of the workshop” = 5.53%, “learning objectives clearly stated” 26.03%, “workshop meets expectation” = 22.26% and “workshop is applicable to clinical practice” = 8.93%.

### 3.4. FASD confidence and knowledge surveys

**Figure 4:**
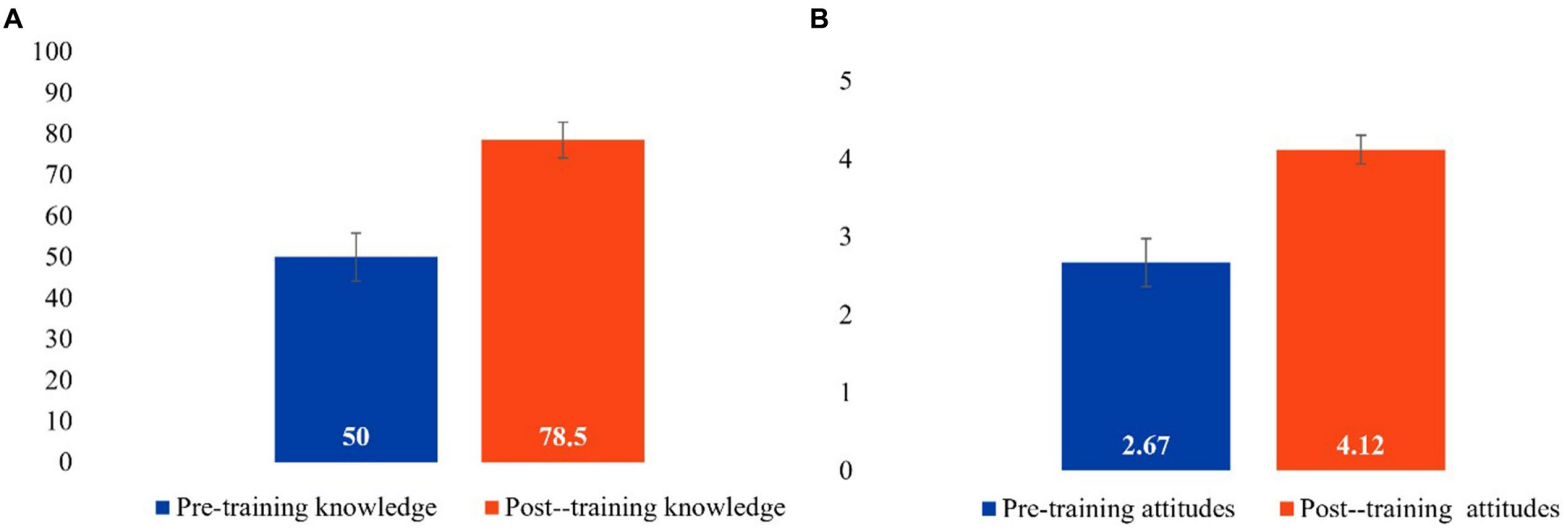
Pre and post training knowledge **(A)** and confidence **(B)**.

A paired-sample *t*-test was used to compare pre and post training clinic knowledge of FASD, and found that FASD knowledge significantly increased from 50.00% pre-training to 78.5% post-training, *t* (19) = −7.14, *p* < 0.001. A separate paired samples *t*-test revealed an increase in participants confidence in FASD knowledge from pre-training (*M* = 2.67) to post-training (*M* = 4.12), *t* (18) = −10.7, *p* < 0.001 (Figure 4).

## 4. Discussion

This project aimed to increase diagnostic service capacity within Australia and improve FASD awareness among health professionals and local communities. Overall, there was a marked increase in FASD diagnostic activity, training activity and community education. Diagnostic activity significantly increased over the course of the project, with over 1,000 individuals assessed, and 425 individuals diagnosed with FASD. The number of FASD diagnoses made by this consortium comprised 81% of FASD cases reported the FASD Australian Registry (FASDAR), and 73% of cases recorded under the National Disability Insurance Scheme (NDIS) as a primary or secondary diagnosis during this reporting period (within the consortium states) (25, 26). Together this suggests that this consortium made a significant contribution to FASD diagnostic activity across Australia during this reporting period. It is acknowledged that the number of FASD cases reported to the FASDAR is likely to be an underestimate, as it requires manual entry by already overburdened clinicians (4), and only captures cases under 15 years old. As part of this consortium, the clinical teams were reminded quarterly to update their FASD cases to FASDAR, and explore methods for embedding this into their regular processes (24).

There was also an increase in community education activity (though not statistically significant). FASD knowledge was also disseminated to over 5,000 attendants across Australia (TAS, WA, NT, SA, VIC), to various community workers, such as education/childcare workers, nurses/midwives, social workers and speech pathologists. The FASD training clinics were successful in increasing FASD knowledge in the attendants, and over 400 clinicians received specialized FASD diagnostic training. Although pre-and post-knowledge surveys were not delivered to the community education session participants, the feedback indicated that participants found the session valuable. In the accompanying qualitative study examining the success of developing FASD models of care at each site (24), each site indicated that one of the key successes was increasing FASD knowledge and interest within their local community. Increasing local knowledge and interest around FASD has the potential to increase FASD prevention, as well as early diagnosis and intervention.

It has been recognized that health professionals play a vital role in FASD prevention efforts (12, 27). Through this study, we demonstrated that a short training session was adequate to significantly increase health professional’s knowledge about FASD. Health professionals have the opportunity to be FASD advocates within the community, and as such, it will be important that they are aware of the lifelong impacts associated with alcohol use during pregnancy, and are consistent with messaging around the impact of prenatal alcohol exposure (16, 28, 29).

Due to the limited diagnostic capacity in Australia (21), it was a key aim of the current study to further train multidisciplinary clinicians to assess and diagnose FASD. It is recognized that although an MDT is the gold standard for assessment and diagnosis of FASD, a more flexible diagnostic approach may be required, which includes further educating a wide range of clinicians, to help provide timely access to services (28). A FASD diagnosis provides an opportunity for individuals and their families to better understand their own strengths and weaknesses and gain access to further support and management for FASD (23, 30). Despite the stigma surrounding FASD as a diagnosis (31, 32), it is important for clinicians to be aware of the positive impact that a FASD diagnosis can have (30, 33). A recent systematic review of the qualitative evidence on FASD lived experience emphasized the impact of receiving a FASD diagnosis (30). The review also highlighted the difficulty in seeking recognition and validation for FASD and related concerns, reinforcing the need for health professionals to receive adequate FASD training.

### 4.1. Limitations and future research directions

This study evaluated the success of rolling out a FASD diagnostic service using a small sample, within a local context, limiting the generalizability of this study. Due to the small sample, we were limited in making inferences about the relative success of each site, though this is further explored in the accompanying qualitative paper (24). Although this study did not directly gather demographic information, most sites were predominantly youth-based. As such, further work is needed to develop appropriate models of care and diagnostic services for adults seeking FASD diagnostic clarity (34). This project focused on improving community knowledge and FASD diagnostic capacity across Australia, however, it is widely acknowledged that further efforts are needed to improve FASD therapy services (35). Future research should also explore the FASD training needs for clinicians, particularly since clinicians attitudes towards FASD (12) and lack of confidence 36 (which is still under review) 37 be a major barrier for diagnosis.

## 5. Conclusion

This paper demonstrated the positive impact of a coordinated approach to FASD prevention, assessment and diagnosis within Australia. Increasing FASD knowledge among clinicians and community members will help to improve FASD prevention and identification efforts.

## Data availability statement

The raw data supporting the conclusions of this article will be made available by the authors, without undue reservation.

## Ethics statement

The studies involving human participants were reviewed and approved by The University of Western Australia Human Research Ethics Committee. The ethics committee waived the requirement of written informed consent for participation in the community education sessions.

## Author contributions

JF and CP conceived the project design and supported the training, opening of the new FASD clinics, and reviewed and edited the manuscript. KP collated and analyzed the data and developed the first manuscript. All authors contributed to the article and approved the submitted version.

## Funding

This work was supported by the Australian Government Department of Health Grant Opportunity—Fetal Alcohol Spectrum Disorder (FASD) Diagnostic Services and Models of Care Project (H1617G038).

## Conflict of interest

The authors declare that the research was conducted in the absence of any commercial or financial relationships that could be construed as a potential conflict of interest.

## Publisher’s note

All claims expressed in this article are solely those of the authors and do not necessarily represent those of their affiliated organizations, or those of the publisher, the editors and the reviewers. Any product that may be evaluated in this article, or claim that may be made by its manufacturer, is not guaranteed or endorsed by the publisher.
