# Supplementary material for: An evaluation of a multi-site fetal alcohol spectrum disorder models of care project
Source: Front Public Health. 2023 Jul 24;11:1195484. doi: 10.3389/fpubh.2023.1195484 (PMC10406497; doi:10.3389/fpubh.2023.1195484)
Supplement: Supplementary file 1 [file Data_Sheet_1.PDF]

**Appendix 1 – Training Clinic Questions**

***Pre-workshop demographic questions***

**Age**:________________ **Gender**:_______________________

**Background, please tick box:**

□ Neuropsychologist
□ Psychiatrist
□ Clinical Neuropsychologist
□ Clinical Neuropsychologist Registrar
□ Clinical Psychologist
□ Clinical Psychologist Registrar
□ Registered Psychologist
□ Psychology Researcher
□ Postgraduate Psychology Student
□ Undergraduate Psychology Student
□ Speech Pathologist
□ Occupational Therapist
□ Paediatrician
□ Physiotherapist
□ Other, please specify:_____________________________________________________

**What age group do you predominantly work with? (Tick all that apply)**

□ Children
□ Adolescents
□ Adults
□ Older adults
□ NA – I do not do clinical work

**How many years have you been working within your profession?:**___________________

***Pre / Post Confidence questions***

**The following questions will help us gain a sense of your pre-existing knowledge of FASD. Please tick the appropriate box for each question. Please do not mark in between numbers or skip any questions.**

| **Question** | **1 Strongly disagree** | **2 Disagree** | **3 Neutral** | **4 Agree** | **5 Strongly agree** |
|---|---|---|---|---|---|
| I have a good understanding of the cognitive features of FASD | | | | | |
| I have a good understanding of the behavioural characteristics of FASD | | | | | |
| I have a good understanding of the social implications of FASD | | | | | |
| I have a good understanding of the psychological characteristics of FASD | | | | | |
| I have a good understanding of the characteristics of FASD as they manifest in children and adolescents | | | | | |
| I have a good understanding of the characteristics of FASD as they manifest in adults | | | | | |
| I am familiar with the Australian Guide to the Diagnosis of FASD | | | | | |
| I am confident in my ability to apply the Australian Guide to the Diagnosis of FASD (as they apply to my discipline) in cases where FASD is a differential diagnosis | | | | | |

| | | | | | |
|---|---|---|---|---|---|
| I have an understanding of the types of treatments and interventions and supports that are helpful for FASD | | | | | |
| I understand the current supports and services which are available to people with FASD in Australia | | | | | |
| I feel confident in my ability to conduct diagnostic assessments with FASD as they apply to my discipline | | | | | |
| I know what the common comorbidities with FASD are | | | | | |
| I am familiar with the common standardised cognitive tests that may be used in FASD assessments | | | | | |
| I am familiar with the common assessments that a paediatrician (or other medical doctor) may conduct in FASD assessments | | | | | |

***Pre/Post knowledge questions***

**Please circle your answer for the following:**

1. The three sentinel facial features are…
   a. Thin upper lip, flat philtrum, short palpable fissure length
   b. Thin upper lip, flat philtrum, long palpable fissure length
   c. Thick upper lip, flat philtrum, short palpable fissure length
   d. Thick upper lip, flat philtrum, long palpable fissure length

2. FASD rarely goes undiagnosed because of its distinctive phenotype.
   a. True
   b. False

3. Which of the following is <u>not</u> an area of impairment in FASD?
   a. Executive functioning
   b. Visuospatial skills
   c. Language
   d. Academic skills

4. In the context of FASD diagnosis using Australian diagnostic guidelines, severe impairment in a neurodevelopmental domain is defined as which of the following?
   a. A score ≥ 1.5 SD below the mean
   b. A score ≥ 2 SD below the mean
   c. A score ≥ 2.5 SD below the mean
   d. A score ≥ 3 SD below the mean

5. According to Australian diagnostic guidelines for FASD, a score at the 2$^{nd}$ percentile on the VCI of the WISC-5 would typically be considered evidence of …
   a. An impairment in expressive language
   b. An impairment in receptive language
   c. An impairment in cognition
   d. All of the above

6. An impairment in working memory would be considered an impairment in which domain?
    a. Memory
    b. Attention
    c. Cognition
    d. Executive functioning

7. According to Australian diagnostic guidelines for FASD, which of the following are considered evidence of severe impairment in affect regulation? (select all that apply)
    a. Oppositional Defiant Disorder
    b. Major Depressive Disorder
    c. Agoraphobia
    d. Post Traumatic Stress Disorder

8. If a person has a preexisting diagnosis of ADHD - Combined Subtype, according to the Australian Guide to the Diagnosis of FASD, this would constitute impairment in which of the following cognitive domains? (select all that apply)
    a. Executive functioning
    b. Affect regulation
    c. Attention
    d. Cognition

9. In Western Australia and the Northern Territory, the rate of FASD is…
    a. About the same in Aboriginal people as in the general population
    b. Lower in Aboriginal people than in the general population
    c. Higher in Aboriginal people than in the general population
    d. Impossible to determine because FASD is thought to be underdiagnosed

10. How much prenatal alcohol exposure needs to occur for FASD to result?
    a. FASD only affects the children of mothers with alcohol dependency
    b. At least one binge drinking episode
    c. More than two standard drinks on any occasion
    d. There is no known safe level of prenatal alcohol exposure

***Post-workshop feedback***

**The following questions will help us to improve future workshops and other training events:**

1) What is your overall assessment of the event? (1 = Insufficient - 5 = Excellent)

1 2 3 4 5

2) Which topics or aspects of the workshop did you find most interesting or useful?

__________________________________________________________________________________________
__________________________________________________________________________________________
__________________________________________________________________________________________
__________________________________________________________________________________________
______________________________

3) Were the learning objectives clearly stated? (please tick box)

□ Definitely
□ Mostly

□ Somewhat
□ Not at all

4) Did the knowledge and information gained from participation at this workshop meet your expectations?
□ Definitely
□ Mostly
□ Somewhat
□ Not at all

5) Will the information covered be useful/applicable in your future clinical practice?
□ Definitely
□ Mostly
□ Somewhat
□ Not at all

6) How do you think the workshop could have been made more effective?
_______________________________________________________________________________________
_______________________________________________________________________________________
_______________________________________________________________________________________
_______________________________________________________________________________________
_______________________________________________________________________________________

7) Comments and suggestions (including activities or initiatives you think would be useful to incorporate in the future):
_______________________________________________________________________________________
_______________________________________________________________________________________
_______________________________________________________________________________________
_______________________________________________________________________________________
_______________________________________________________________________________________

8) What did you like most about the workshop?
_______________________________________________________________________________________
_______________________________________________________________________________________
_______________________________________________________________________________________
_______________________________________________________________________________________
_______________________________________________________________________________________
